# Tap Water vs. Sterile Saline for Irrigation of Traumatic Soft-Tissue Wounds in the ED

**DOI:** 10.7759/cureus.98790

**Published:** 2025-12-09

**Authors:** Henry Mills, Nikhil Pandit, Lucky Jeyaseelan, Amit Patel

**Affiliations:** 1 Barts Bone and Joint Health, The Royal London Hospital, Barts Health NHS Trust, London, GBR

**Keywords:** acute traumatic wound, healthcare efficiency, hospital tap water, irrigation, low-cost healthcare, medical waste, tap water, washout, waste management, wound infection rate

## Abstract

Traumatic wounds are a common presentation in EDs and are routinely irrigated to reduce infection risk. Sterile saline (SS) is traditionally used, though tap water (TW) may offer advantages in cost, availability, and environmental sustainability. A narrative review was conducted using PubMed and Google Scholar (August 2025) to identify systematic reviews, randomized controlled trials, observational studies, and guidelines comparing TW and SS for traumatic wound irrigation in emergency settings. Outcomes assessed included infection rates, cost, environmental impact, workflow efficiency, and accessibility. Evidence from randomized trials and a recent systematic review demonstrates no significant difference in infection rates between TW and SS, with some studies favoring TW. These findings, combined with the considerable reductions in cost and environmental impact, along with the greater practicality of TW, suggest it is a safe and effective alternative when potable water is available. Remaining concerns relate mainly to water quality and clinician acceptance, particularly in regions where water quality is variable. Additional multicenter high-quality studies are needed to clarify any absolute infection differences and to explore TW implementation across diverse clinical and resource contexts.

## Introduction and background

Traumatic wounds are among the most common presentations to EDs, accounting for approximately 5% of attendances globally [1-3]. Infection is the most common complication in these patients (3-5%) [1,3]. Early wound irrigation is a well-established component of acute wound management, shown to reduce infection risk and promote healing [1]. It achieves this by removing debris, reducing the bacterial inoculum (the concentration of bacteria within a wound), and optimizing the wound environment for healing [4].

The significance of the bacterial inoculum has been highlighted by studies showing that wound infections are strongly associated with bacterial concentrations of ≥10⁶ [5]. Both animal and human studies demonstrate that irrigation significantly reduces bacterial counts and subsequent infection rates [6-8]. Timing of irrigation is also critical. In one animal model of contaminated wounds, washout reduced bacterial counts by 70%, 52%, and 37% when performed at three, six, and 12 hours, respectively [4]. The phrase “the solution to pollution is dilution” is frequently used in the clinical setting to emphasize the importance of irrigation [9]. National guidelines around the world universally reflect this consensus, recommending early wound washout in the ED [10].

In contrast, the approach to open fracture wounds is less consistent. For example, the British Orthopaedic Association Standards for Trauma (BOAST 2017) guidelines advise against wound washout before theater, citing the benefits of surgical sterility, specialist expertise, and controlled equipment use when managing these more complex wounds with a higher risk of complication [11]. However, this stance is debated, as some experts argue that delaying irrigation allows bacterial contamination to progress and that early ED decontamination may reduce infection risk. The recent International Consensus Meeting on Musculoskeletal Infection recommended that initial irrigation should be performed as early as possible in the ED, rather than delaying until operative debridement [11].

When choosing the washout solution, most guidelines recommend sterile saline (SS), tap water (TW), or the option to use either [10,12-14] (Table 1). However, surveys and observational audits (mainly in the United Kingdom, Australia, and North America) show SS continues to be the predominant irrigant for traumatic wounds [15,16]. This preference appears to be driven more by habit, perceived sterility, and concerns about legal or professional scrutiny than by evidence of superior clinical outcomes [15,16]. Nevertheless, some guidelines, such as those from the American College of Emergency Physicians (ACEM), explicitly endorse TW, highlighting its safety, lower costs, greater availability, reduced waste production, and practical administration [14,16,17].

**Table 1:** Key guidelines and their solution recommendations for acute traumatic wounds ACEM, American College of Emergency Physicians; ACI, Agency for Clinical Innovation; CKS, UK Clinical Knowledge Summaries; NICE, National Institute for Health and Care Excellence; PIC, Paediatric Improvement Collaborative; SS, sterile saline; TW, tap water

Guideline	Recommendation	Reference
Local PIC-endorsed guidelines in Australia, including Perth Children’s Hospital and Royal Children’s Hospital, Melbourne	TW or SS	[10]
ACI, New South Wales Health, Australia, for adults and pediatrics	TW or SS	[12]
NICE/CKS	TW or SS	[13]
United States - ACEM	TW	[14]

Given the lack of consensus on irrigation solution choice and the substantial cost and waste implications of using single-use plastics, this article evaluates the current evidence comparing TW and SS for traumatic soft-tissue wound irrigation in the ED. It focuses primarily on infection rates while also considering financial, practical, and environmental factors. Open fractures are excluded due to the ongoing debate around whether irrigation should be performed prior to the operating theater. Additional emphasis is placed on randomized and quasi-randomized controlled trials (RCTs), with supporting microbiological and cost analyses included where relevant.

## Review

Methods

This narrative review aims to summarize the comparative evidence on the use of TW versus SS in wound irrigation. Studies were identified through targeted searches of PubMed and Google Scholar in August 2025 using combinations of the terms “tap water”, “sterile saline”, “wound irrigation”, and “infection”. Additional papers were located by screening the reference lists of key articles and previous reviews. Animal studies initially identified were excluded from the final synthesis, as their relevance to ED practice is limited and they do not contribute meaningful clinical guidance. The focus is on prospective comparative human studies that reported infection outcomes or bacterial concentrations in acute traumatic wounds. The human trials included in this review predominantly enrolled uncomplicated acute traumatic wounds such as lacerations and abrasions. High-risk or complex wounds such as bites, puncture wounds, heavily soiled wounds, foreign bodies, burns and other thermal injuries, and open fractures were typically excluded.

No formal systematic search strategy or predefined inclusion or exclusion criteria were used. As such, this review does not follow the Preferred Reporting Items for Systematic reviews and Meta-Analyses (PRISMA) methodology. This introduces a risk of selection bias and the possibility that relevant studies may not have been captured. The findings should therefore be interpreted with appropriate caution.

To contextualize clinical practice and health-economic considerations, national guidelines, procurement information, and NHS sustainability reports were also reviewed. These sources were used to illustrate real-world practice variations, cost implications, and environmental impact.

Infection

A total of eight comparative studies were included in this narrative synthesis: one systematic review, which conducted pooled analyses for only a subset of outcomes due to study heterogeneity; six RCTs in humans; and one prospective cohort study.

Evidence from observational studies, randomized RCTs, and systematic reviews consistently reports similar infection rates with wound irrigation using TW and SS [1,3,15,18-21]. The three largest and most methodologically robust RCTs each enrolled between 625 and 705 patients with acute traumatic wounds in EDs [1,3,19]. With baseline infection rates of 3-6%, these trials were powered to detect absolute differences of approximately 5-6%, meaning they could exclude any clinically meaningful advantage of one solution over the other rather than demonstrate absolute equivalence [22]. Moscati et al. and Weiss et al. used double-blind designs, while Angerås et al. included a broader spectrum of wounds, enhancing external validity [1,3,19]. Although specific definitions varied between studies, infections were consistently defined using objective criteria such as purulent discharge, delayed healing, multiple signs of inflammation, or the need for a significant change in management (e.g., antibiotics, debridement, or early suture/staple removal). Infection rates across these studies were low and generally similar between groups (typically 3-6%), with no significant differences in wound healing or adverse outcomes [1,3,19]. However, Angerås et al. reported a significantly lower infection rate with TW compared with SS (5.4% vs. 10.3%; p < 0.05) [19]. This may reflect differences in wound selection and methodology rather than a true biological effect, as the trial included a wider range of wound types and did not stratify by contamination level. These trials also excluded high-risk wounds and immunocompromised patients and were conducted in high-income regions with reliably potable water, limiting broader applicability [1,3,19].

Additional RCTs likewise found no difference in infection rates but were underpowered (n < 60) [18,20,21]. A related RCT by Resende et al. compared bacterial load reduction in 120 mixed wound types [23]. They observed similar reductions in bacteria and fungi between TW and SS, but greater gram-positive clearance with TW (p = 0.041) [23]. However, the timing between irrigation and post-irrigation sampling was not reported, making it unclear whether contaminants in TW would have had sufficient time to inoculate the wound. Moreover, the heterogeneity of wound etiologies limits interpretation [23]. The exact figures and details of these studies are presented in Table 2.

**Table 2:** Human trials comparing infection rates and bacterial counts: TW vs. SS RCT, randomized controlled trial; SS, sterile saline; TW, tap water

Study	Year	n	Key finding	Study design	Limitations
Moscati et al. [1]	2007	634	Infection: 4% TW vs. 3.3% saline	Double-blind RCT	Urban single-center (Buffalo, USA)
Weiss et al. [3]	2013	625	Infection: 3.5% TW vs. 6.4% saline	Double-blind RCT; most standardized trial	Excluded high-risk wounds; adult ED only
Griffiths et al. [18]	2001	49	No significant difference	Double-blind RCT	Small sample size
Angerås et al. [19]	1992	705	Infection: 5.4% TW vs. 10.3% saline	Large quasi-RCT; pragmatic real-world population with a spectrum of wounds	No blinding; single-center
Valente et al. [20]	2003	60	No difference in infection or healing	Quasi-RCT	Underpowered
Bansal et al. [21]	2002	46	No difference	Prospective cohort study	Small sample size; limited external validity
Resende et al. [23]	2015	120	TW ↓ Gram-positive bacterial counts	Double-blind RCT; microbiological data	Heterogeneous wound types

The most comprehensive synthesis comes from the Cochrane review by Fernández et al. [15]. The 2022 update included eight RCTs (n = 2,204) comparing TW and SS for irrigation of a variety of acute and chronic wounds. Pooled analysis showed a non-significant 16% relative reduction in the risk of infection with TW (risk ratio 0.84, 95% CI 0.59-1.19), and subgroup analyses for both acute and chronic wounds demonstrated the same direction of effect [15]. However, the evidence was downgraded to “low certainty” due to heterogeneity between studies, variation in irrigation technique and water source, limited blinding, and imprecision from low event rates. Furthermore, all trials were conducted in settings with potable water and largely excluded high-risk or immunocompromised patients, limiting global applicability [15].

Taken together, the trials reviewed suggest that when potable TW is available, it is as safe as SS for wound irrigation, with no reported serious or long-term adverse outcomes in these studies. However, these findings apply only to environments where TW quality is reliably potable, and the certainty of no absolute difference in infection rates remains low.

Wider impacts

Given that SS offers minimal or no advantage in preventing infection, other considerations, such as cost, waste and sustainability, workflow efficiency, patient experience, and accessibility, become highly relevant.

Cost

Moscati et al. conducted a detailed cost comparison between SS and TW irrigation in a US medical center [1]. The supply charge for 500 mL of saline was US$9.11 (saline US$3.08; syringe US$0.90; splash shield US$5.13), versus approximately US$0.0015 for TW (plus US$0.60 for extension tubing in 36% of cases) [1]. When extrapolated to an estimated 8 million annual laceration repairs, and factoring in infection outcomes and treatment costs, switching to TW was projected to save US$65.6 million (∼90% reduction) [1]. These calculations used the upper confidence interval for TW infection rates, making the estimate conservative.

However, the analysis reflects US pricing and cannot be directly applied to other health systems without local cost assessments. It also likely underestimates the true savings, as it excludes additional SS-related costs such as multiple saline bottles, syringes, splash shields, procurement, storage, staff time, and clinical waste disposal.

Waste and Sustainability

Replacing single-use SS with potable TW would also reduce the environmental and financial burden associated with clinical waste, particularly given the NHS commitment to achieving net-zero emissions by 2040 [24]. The NHS in England generates approximately 156,000 tons of clinical waste annually, much of which requires energy-intensive transport and incineration. Incineration alone costs around £617 per ton, equating to over £32 million per year in disposal costs [24]. According to NHS sustainability guidance, clinical waste incineration produces approximately 1 ton of carbon dioxide emissions per ton of waste [24]. Reducing the use of single-use plastics for irrigation could therefore provide meaningful cost and carbon savings, aligning with NHS sustainability objectives.

Workflow Efficiency

From a practical standpoint, TW offers significant workflow and accessibility advantages in both high- and low-resource settings. It requires no sterile packaging, storage, procurement, or disposal. It is immediately available at the point of care, and in busy EDs, this may reduce small but cumulative delays associated with sourcing SS and preparing syringe-based irrigation. Douglas and Cornish suggested that TW cleansing could be patient-directed at the sink, which could ultimately free up staff for other tasks and improve departmental efficiency [16].

Patient Experience

Two large RCTs conducted in Sweden and the USA compared patient-reported comfort and satisfaction using TW versus SS for wound irrigation, finding no significant differences in pain, acceptability, or overall satisfaction [1,19]. Furthermore, satisfaction may be higher when the healthcare provider does not need to be in such close proximity, reducing splatter risk and body-fluid contamination [1].

Accessibility

Globally, approximately 74% of the population now has access to safely managed potable water, making TW irrigation feasible across most high- and many middle-income regions [25]. However, Fernandez et al. noted the absence of evidence for TW irrigation in areas without potable water and cautioned that safety cannot be assumed where water quality is uncertain [15]. In low-resource or emergency settings, where SS may be unavailable or prohibitively expensive, the option to use reliably potable TW would offer advantages for global health equity; however, in many such settings, TW quality may be inadequate. Conversely, in regions with water scarcity, such as drought-affected areas with hosepipe bans, the availability and practicality of TW irrigation must be considered within the local context.

Discussion

The literature consistently supports potable TW as a safe and effective wound irrigation solution in the ED [1,3,7,15,18-21,23]. Across multiple RCTs, infection rates with TW are comparable, and in some cases superior, to those with SS, making a true infection-prevention advantage of SS unlikely [1,3,18-20]. Although not definitively established, several authors have proposed plausible explanations for why some studies observed lower infection rates with TW. TW irrigation typically involves higher flow rates and larger delivered volumes compared with syringe-based saline irrigation, which may enhance mechanical removal of bacteria and debris [1,3]. In addition, TW is often delivered at a comfortable lukewarm temperature, potentially improving patient tolerance and enabling more thorough cleansing. These technique-related differences, rather than any intrinsic antimicrobial effect, may explain the direction of effect observed in some trials.

The strength of conclusions regarding absolute differences remains limited by underpowered studies and methodological variability. Studies differ widely in irrigation technique, pressure, temperature, and volume, complicating meaningful comparison and meta-analysis [15,16]. Moreover, many studies excluded high-risk wounds and immunocompromised patients, limiting generalizability to broader emergency populations [1,3]. Moscati et al. also highlighted the practical challenges of adequately powering wound irrigation trials, given low infection event rates, slow recruitment, and protocol variability [1]. Taken together, these limitations mean that the evidence supporting clinical equivalence between TW and SS remains low-certainty, and any practical or economic advantages should be interpreted in this context. Future large, multicenter trials with standardized protocols, including irrigation solution, volume, technique, and temperature, are needed to provide more definitive evidence. Such studies should also evaluate long-term outcomes, cost-effectiveness, and patient satisfaction across diverse healthcare systems to determine not only clinical equivalence but also real-world sustainability and acceptability.

Despite these limitations, the collective evidence still leans toward TW as a safe alternative. Logistically, TW irrigation may also improve efficiency by allowing earlier initiation of wound cleansing, such as at triage, potentially reducing waiting times and improving patient flow [16]. In recognition of this growing body of evidence, the ACEM formally endorsed hospital TW as an acceptable wound irrigant in 2024, citing its safety, efficacy, and environmental and cost benefits [14]. However, barriers to the widespread adoption of TW remain, as clinician preference and institutional protocols often favor SS, reflecting a historical assumption of its superiority [15,16]. As an interim step while further trials are awaited, high-resource EDs with reliably potable TW could reasonably conduct local pilot implementations to quantify setting-specific cost, waste, and workflow impacts using TW for simple wounds, thereby validating these secondary benefits within their own systems.

## Conclusions

When reliably potable, TW appears to be a safe, practical, and cost-effective alternative to SS for wound irrigation in the ED. Comparative studies have not shown an increased risk of infection with TW. Broader adoption could result in substantial reductions in healthcare costs, environmental waste, and reliance on single-use sterile products.

However, these conclusions should be interpreted cautiously, as the evidence base remains low-certainty due to small sample sizes, methodological heterogeneity, and limited data from low-resource settings. Paradoxically, such settings, where SS supplies are most constrained, may also have the least reliable access to truly potable water. Further multicenter research is needed to clarify any absolute differences in infection rates and to quantify the wider impacts of TW adoption across diverse clinical and resource settings.

## References

[1] Moscati RM, Mayrose J, Reardon RF, Janicke DM, Jehle DV (2007). A multicenter comparison of tap water versus sterile saline for wound irrigation. Acad Emerg Med.

[2] Prevaldi C, Paolillo C, Locatelli C, Ricci G, Catena F, Ansaloni L, Cervellin G (2016). Management of traumatic wounds in the emergency department: position paper from the Academy of Emergency Medicine and Care (AcEMC) and the World Society of Emergency Surgery (WSES). World J Emerg Surg.

[3] Weiss EA, Oldham G, Lin M, Foster T, Quinn JV (2013). Water is a safe and effective alternative to sterile normal saline for wound irrigation prior to suturing: a prospective, double-blind, randomised, controlled clinical trial. BMJ Open.

[4] Owens BD, Wenke JC (2007). Early wound irrigation improves the ability to remove bacteria. J Bone Joint Surg Am.

[5] Raju DR, Jindrak K, Weiner M, Enquist IF (1977). A study of the critical bacterial inoculum to cause a stimulus to wound healing. Surg Gynecol Obstet.

[6] Badia JM, Torres JM, Tur C, Sitges-Serra A (1996). Saline wound irrigation reduces the postoperative infection rate in guinea pigs. J Surg Res.

[7] Moscati R, Mayrose J, Fincher L, Jehle D (1998). Comparison of normal saline with tap water for wound irrigation. Am J Emerg Med.

[8] Mueller TC, Mehraein N, Kehl V, Dimpel R, Friess H, Reim D (2025). Antiseptic wound irrigation to prevent surgical site infection after laparotomy: meta-analysis. BJS Open.

[9] Singer AJ, Dagum AB (2008). Current management of acute cutaneous wounds. N Engl J Med.

[10] (2025). Lacerations. https://www.rch.org.au/clinicalguide/guideline_index/Lacerations/..

[11] Sandean D (2021). Open fractures - what is the evidence, and how can we improve?. Arch Bone Jt Surg.

[12] (2025). Minor wounds. https://aci.health.nsw.gov.au/ecat/adult/minor-wounds.

[13] (2025). Scenario: Management | Management | Lacerations | CKS | NICE. https://cks.nice.org.uk/topics/lacerations/management/management/..

[14] (2025). Timely council resolution encourages using tap water for wound irrigation. https://www.acep.org/news/acep-newsroom-articles/timely-council-resolution-encourages-using-tap-water-for-wound-irrigation.

[15] Fernandez R, Green HL, Griffiths R, Atkinson RA, Ellwood LJ (2022). Water for wound cleansing. Cochrane Database Syst Rev.

[16] Douglas H, Cornish L (2016). Cleansing of acute traumatic wounds: tap water or normal saline?. Wounds UK.

[17] Holman M (2023). Using tap water compared with normal saline for cleansing wounds in adults: a literature review of the evidence. J Wound Care.

[18] Griffiths RD, Fernandez RS, Ussia CA (2001). Is tap water a safe alternative to normal saline for wound irrigation in the community setting?. J Wound Care.

[19] Angerås MH, Brandberg A, Falk A, Seeman T (1992). Comparison between sterile saline and tap water for the cleaning of acute traumatic soft tissue wounds. Eur J Surg.

[20] Valente JH, Forti RJ, Freundlich LF, Zandieh SO, Crain EF (2003). Wound irrigation in children: saline solution or tap water?. Ann Emerg Med.

[21] Bansal BC, Wiebe RA, Perkins SD, Abramo TJ (2002). Tap water for irrigation of lacerations. Am J Emerg Med.

[22] Chow SC, Shao J, Wang H, Lokhnygina Y (2017). Sample Size Calculations in Clinical Research, 3rd Edition. Chapman and Hall/CRC: New York.

[23] Resende MM, Rocha CA, Corrêa NF (2016). Tap water versus sterile saline solution in the colonisation of skin wounds. Int Wound J.

[24] (2025). NHS clinical waste strategy. https://www.england.nhs.uk/long-read/nhs-clinical-waste-strategy/.

[25] Johnson B (2025). Progress on household drinking water, sanitation and hygiene 2000-2024: special focus on inequalities. https://data.unicef.org/resources/jmp-report-2025/.

